# Experiences related to health promotion behaviors in overweight pregnant women: a qualitative study

**DOI:** 10.1186/s12978-018-0660-y

**Published:** 2018-12-29

**Authors:** Azita Fathnezhad Kazemi, Sepideh Hajian

**Affiliations:** 1 Student Research Center, School of Nursing and Midwifery, Shahid Beheshti University of Medical Sciences, Tehran, Iran; 2 Department of Midwifery & Reproductive Health, Faculty of Nursing & Midwifery, Shahid Beheshti University of Medical Sciences, Tehran, Iran

**Keywords:** Health promotion, Pregnancy, Lifestyle, Overweight, Qualitative study

## Abstract

**Background:**

The adoption of health behaviors by pregnant women causes their improved health and achievement of appropriate pregnancy outcomes as well as improving the quality of life of the mother and infant. Limited studies have examined such behaviors in pregnant women, especially in overweight mothers. The purpose of this study was to explore the experiences of overweight pregnant women in relation to lifestyle changes during pregnancy to improve their health.

**Methods:**

This qualitative study was carried out with content analysis approach in Tabriz-Iran in 2017. Using purposive sampling, pregnant women with the Body Mass Index of overweight at the preconception were selected considering other inclusion criteria and the sampling was continued to saturate the findings. The data were collected by the first author through semi-structured face-to-face interviews with 32 overweight pregnant women through 18 deep individual interviews and three group interviews. The MAXQDA software version 10 was used for data analysis. Data strength was confirmed by participants and external control.

**Results:**

Most participants were Primigravida 20 (62.5%) and with high-level education 25 (78.1%). Qualitative data analysis led to the emergence of three main themes: “physical self-care”, “mental self-care”, and “risk perception”. The first two themes present the nature of health promotion behaviors in overweight pregnant women; the third theme expresses their feelings and perception of behaviors related to health promotion.

**Conclusion:**

Health promoting behaviors include behavioral and cognitive actions resulting from a perception of the risk caused by overweight pregnant mothers. Therefore, pregnancy is the right time to evaluate behaviors and to use high motivation of women to guide them in choosing healthy behaviors and helping them to adhere to health related behaviors.

## Plain English summary

Many diseases are caused by unhealthy lifestyle; pregnancy period is considered critical because of the impact of maternal behaviors on the consequences of childbirth and the quality of life of mother and child. Nowadays, due to lifestyle changes, half of women are overweight when their pregnancy begins; while limited information is available on health-related behaviors such as nutritional behaviors, physical activity status, and other health promoting behaviors in pregnant women, especially overweight ones. Since awareness of these behaviors will be helpful in planning interventions in this study, it was tried to interview with 32 overweight pregnant women to examine their experiences and their performance in promoting health behaviors. Data analysis showed that changing health-related behaviors in pregnant women is due to the perception of the health risks of the child and the existence of such an emotion increases the incentive to make positive changes in everyday behaviors which are in the form of increasing the attention to and practice of actions in the field of physical and metal self-care, such as improving nutritional behaviors, performing health behaviors, changing sleep and waking patterns, paying attention to pregnancy changes and needs and adopting stress control methods, including trying to avoid stressors, having social interactions and seeking social support. The ultimate goal of doing such behaviors is the birth of a healthy baby.

## Background

Health promoting behaviors are a way to maintain and improve health [1–3]. The impact of behaviors and how pregnant women treat with different situations of life on maternal and neonatal outcomes are not overlooked [4, 5]. The adoption of a health promoting lifestyle during pregnancy can be a safe strategy to maintain and improve maternal and child health [2, 6]. Such behaviors include the adoption of appropriate ones in various aspects of lifestyle that leads to improved physical and mental health [5, 7] and the prevention of chronic diseases [8] so today, due to the importance and potential role of lifestyle in the development of chronic diseases, attention to health promotion behaviors for maintaining and improving the health status has significantly increased [9].

Over the past two decades in the world, we have seen an increase in obesity and overweight in women of childbearing age and pregnant women [10, 11]. According to reports, 50% of women in developed countries have a Body Mass Index of over 25 at the beginning of pregnancy [12, 13]. Rising national incomes in developing countries and increased `Westernization’ lead to increased levels of overweight and obesity in the women [14]. However, these women have an unhealthy lifestyle such as inappropriate nutrition and sedentary lifestyles [15]. However, the adaptation of the pregnant mother to the changes in various aspects of life is important, and searching for safe and healthy behaviors is considered to be a native task. [2, 3, 16]. Looking for safe and healthy behaviors is considered to be from the maternal tasks [5, 16]. The adoption of health-related behaviors in various aspects of life during pregnancy, including control of weight gain, ability to control stress, and participation in social groups can have direct and indirect effects on pregnancy outcomes [17, 18]. The results of a systematic review of such behaviors in pregnancy indicated that based on numerous quantitative studies, not only pregnant women obtained moderate scores for performance in the overall score of health promotion behaviors and high scores were related to the dimension of spiritual growth (self-actualization) and nutritional status, but also several factors like race, culture, religion, self-care, efficacy can affect their performance [16].

In previous studies, experiences related to health promotion behaviors have been studied in different groups such as women of reproductive age [19, 20], as well as female students [21]; but studies in pregnant women are limited. In some studies, concerns and experiences related to health behaviors have been investigated in pregnant women with high-risk pregnancies, including pregnant women over 35 years of age [22], addiction [23, 24], and obesity [25, 26]. Several reasons have caused this study to be designed with the aim of explaining the experiences of health promotion behaviors in overweight pregnant women. These reasons include: the conditions and factors affecting health vary from country to country; planning for health-medical and psychosocial services requires the discovery and interpretation of the viewpoint of high-risk groups such as pregnant mothers; and finally, the lack of qualitative research on the concept of health promoting of overweight pregnant mothers, which can have appropriate efficiency in explaining concepts and understanding phenomena such as health, with emphasis on social context so we aimed to examine experiences health promoting holistically in overweight pregnant women for designing appropriate interventions in preventing Obesity and managing weight gain in pregnancy.

## Methods

### Study design and participants

This qualitative study was conducted with the content analysis approach from April to September 2017 in Tabriz-Iran. Iran has different ethnic groups [27]. Tabriz is the fifth largest city in Iran with a population of about one and half million [28] with more than 30,000 births occur annually [29] also the majority of them speak in Turkic languages [28].

The inclusion criteria of the study included single pregnancy with the age range of 18–40 years, having the language skills, pregnancy over 10 weeks, and having information about weight and height preconception in the women’s health records whom are defined as overweight according to their BMI (25–29.9). Having any disease or medical, midwifery risk factor identified on the basis of the family’s health records or, as the pregnant woman claims, also requiring special care or relative or absolute rest for the mother, as well as the withdrawal and non-attendance of the mother were considered as the exclusion criteria. The purposive sampling was used for individual and group interviews with pregnant women referring to health care centers or pregnant women’s care clinics.

All individual and group interviews were conducted by the original author of the paper (A.Fnk) as a faculty member and a Ph.D. student in reproductive health who has a history of participation in the classes of qualitative research methodology and the use of qualitative analysis software also she works as a midwife in prenatal care. All steps for data recording and data analysis were conducted under the supervision of the author (S.H) as a reproductive health faculty member with several years of qualitative study.

### Data collection

After selecting people according to the inclusion criteria of the study through the case of pregnant mothers, at first, in an acquaintance or telephone meeting the purpose and reasons of the study were explained to each of the participants and, according to the willingness of the participants, the time of in-person interviews was determined. Initially, five interviews were conducted in pilot form; these interviews were not included in the analysis. Semi-structured questions were used to conduct interviews. These questions were set by reviewing the texts and based on the experience of the author responsible for qualitative studies. We followed Pender’s Health Promotion Mode and according to prior researches on health promotion, at first, open questions were asked (Table 1: Interview questions). Then interviews focused on behaviors were expressed based on the answer to each question. Participants were also asked to provide more detailed explanation of the issues or to express their purpose more precisely. To record the data, interviews were first recorded and Immediately after each interview, the text was typed. As far as possible, the Note field was used and non-verbal data such as tone of voice and behaviors were recorded, too. Sampling was carried out in pregnant women’s care centers in a quiet room and without someone other than the interviewees. Each participant was given the code and nickname. Interviews lasted from 30 to 60 min. Interviews continued until data saturation, which was achieved in the 14th interview. Nevertheless, two other interviews were conducted to ensure that it was completed. A total of 16 individual interviews and three group interviews (two 5-person interviews and one 6-person interview) were conducted. A total of 32 people participated in the interviews.

**Table 1:** Questions used to promote discussion in focus groups and interview

- What do you mean by health?	
-What behaviors and factors improve the health of the person?	
- How do you define health and health improvement concepts during pregnancy?	
-What do you do as a pregnant woman to maintain and improve your health during this period?	
-What factors make pregnant women more motivated to do behaviors that increase their health?	
-Suppose I’m a pregnant woman; what recommendations will I make to improve my health?	

Two of the interviews were discontinued because of the participants’ dissatisfaction with the continuation of the interview. In one case, a 19-year-old participant was excluded from the study due to she’s inability to answer the questions, and in the latter case, because of the mother’s dissatisfaction and frustration to continue the interview. One of the interviews was interrupted due to ambient noise and was repeated a few days later.

### Data analysis

Data analysis process was performed simultaneously with data collection using MAXQDA software version 10.

Data analysis was performed using content analysis with a conventional approach based on the Graneheim and Lundman method [30]. Identified codes are the result of semantic units from the participants’ descriptions. The codes were categorized into themes and main categories. Subcategories and Sub-subcategories were also extracted based on differences and similarities (Table 2: Examples of content analysis, coding, and categorization).

**Table 2:** An example of analysis process

Meaning unit	Code	Sub-Subcategory	Subcategory	Main Category	Theme
I studied the Internet and books and made a diet for myself	Weight control measures in pregnancy	Interest in changing behavior	Behaviors according to new needs	Modification of lifestyle	Physical self-care
Now (in pregnancy) I’m doing walking or tensile exercise that I learned in physiological delivery classes.	Conducting specific exercises during pregnancy				
I was wearing tight clothes, I’m not wearing them now, I’m wearing loose clothes; I do not get annoyed, when I’m so warm loose clothes are good for me	Changes in apparent and behavioral habits	Force to change behavior			
Before pregnancy, I could sleep and eat as I wanted, go, drive, and could exercise at every hour; but now, I’m not doing it anymore. I do care about what I’m doing. For example, before I got pregnant, I run in the street when it was late, but now I do not have to run.	Decreasing some of favorite activities before pregnancy				
My sexual intercourse has become less and limited and I become more cautious with higher stress. (Laughs) But I think it’s principled	Changing sexual activity				
The nuts, for example, I use more nuts because I was afraid of obesity and it was all in the diet I avoid eating, but now, we completely put the diet down.	Elimination of slimming diet before pregnancy				
I’m shutting down things that are hard and harmful to me. I do not carry anything heavy. Well, I do not lift up a lot of things, or try to do homework alone.	Avoiding Hard and Tedious Activities				

### Validation

To validate the results, it was first tried to establish a friendly relationship with the participants. In order to increase the accuracy of the data and for verification of the accuracy of the data, after the registration, the interviews were given to the participants to review and confirm their stated content and, if there were any other content, it has been added to the data. Interviews were frequently read by the corresponding author of the paper; then, the text of the interviews with the extracted codes and categories was shared with the colleagues and their comments were used. External monitoring was also used to increase the reliability. By providing the initial code derived from the analysis and examples of the extraction, as external observers, the concepts were given to four reproductive health researchers who were not related to the study in order to determine whether they also had a similar perception of the data or no.

## Results

The analysis of demographic characteristics indicated that, the majority of pregnant mothers were 19 (59.4%) in the age range of 25 to 34 years old, 25 (78.1%) had high education and more than two thirds were housewives. Also, the majority of them 20 (62.5%) were Primigravida and all of them live in urban areas. (Table 3: Demographic characteristics of participants).

**Table 3:** Sociodemographic characteristics of participants

Variable	Mean (SD)
Age (in years)	29.5 (5.04)
Pre-pregnancy BMI	27.54 (1.37)
Number of pregnancies	1.5 (0/97)
Variable	Number (Percent)
Education	
Elementary	2 (6.3)
Secondary	5 (15.6)
High school	9 (28.1)
University	16 (50.0)
Occupation	
Housewife	25 (78.1)
Employed	7 (21.9)
Spouse’s education	
Elementary	3 (9.4)
Secondary	5 (18.3)
High school	11 (34.4)
University	13 (40.6)
Spouse’s occupation	
Employee	5 (15.6)
Worker	7 (21.9)
Self-employed	20 (62.5)
Family structure	
Nuclear	30 (93.7)
Extended	2(6.3)
Adequacy of income	
At a sufficient level	10 (32.2)
Less than adequate	22 (68.8)
Ethnic group	
Azerbaijanis	31 (96.8)
Persians	1 (3.2)

In the process of analyzing qualitative data after categorizing codes and removing similar codes, 106 codes were classified in 34 sub-subcategories, 15 subcategories, 7 main categories and 3 themes of “physical self-care”, “mental self-care”, and “risk perception” (Table 4: Classification of Them).

**Table 4:** Classification of Them, main categories and subcategories

Theme	Main categories	Subcategories	Sub- Subcategories
Physical self-care	Modification of lifestyle	Improving Nutrition Behaviors	Pay attention to the quantity of nutrition
			Pay attention to the quality of nutrition
			Pay attention to timing and habits of food consumption
		Improved controllable health behaviors	Pay attention to individual factors
			Attention to environmental hazards
		Change the sleep and awakening pattern	Pay attention to sleep hygiene
			Need more rest
	Adopting new behaviors	Behaviors that meet new needs	Interest in changing behavior
			Force to change behavior
		Adopting preventive behaviors	Doing recommended behaviors
			Behaviors learned
		Adopting avoidance strategies	Distracting thoughts
			Pretending
Mental self-care	Stress management strategies	Solution-based strategies	Control / elimination of stressors
			Trying to cope with stress
		Positive coping strategies	Thinking positive
			Evolution
			Spirituality
	Tendency to social interactions	Tendency to join the group	Interact with counterparts
			Being with your loved ones
	Searching for social support	Search for psycho-emotional support	Husband support
			Family and friends support
			Health care support
		Search for information and tool support	Tool support by husband and allies
			Health care information support
			Legislators/policy makers support
Risk perception	perception of the threat	Perception of Health Sensitivity	Sensitivity to fetal/infant health
			Mother Health Sensitivity
		Perception of the pregnancy outcomes	Consequences of pregnancy
			Cultural pressures
	controlling the threat	Trying to pass healthy pregnancy	Understanding the Importance of Health in Pregnancy
			Efficient measures
		Reducing risk factors	Trying to give birth to a healthy baby
			Trying to maintain health

### Physical self-care

This theme was classified in two main categories, namely, the modification of the lifestyle and the adoption of new behaviors extracted from five categories and eleven subcategories.

### Modification of lifestyle

Modification of lifestyle is a major category that reflects the attention of pregnant mothers to various aspects of lifestyle and attempts to change to improve the status quo. This main category was derived from the sub-categories of improving nutritional behaviors, modifying health behaviors and the pattern of sleep and rest.

The quantity and quality of food consumed were considered more in adopting a proper nutritional pattern; so that most participants mentioned increasing consumption of natural and beneficial foods and prefer the consumption of milk and dairy, fruits and vegetables, and avoid consumption of prepared foods, sugars, and fats and salts, and strive for balanced consumption of food groups and attention to religious teachings related to food intake and timely meals. For example, concerning the quantity, quality, and eating habits of participants, they said:

*“I did not eat vegetables much (laughing), for example, I ate them once a week, but now that I’m pregnant, I eat vegetables every day at lunch and dinner without exception. I pay much attention to my nutrition, for example, I used to eat a lot of salt before. I do not get too much salt now. Pizza, sausages, I liked and ate fried foods a lot, but now I rarely consume them.”(p.1).*

*“When I’m eating, I’m caring for chewing food too much and eating food slower. In addition, I also pay attention to my nutrition. For example, I’ll not start the day without breakfast; while previously, I could not eat anything from morning to noon, but now I’m sure to have breakfast.” (p.2).*

*“Before pregnancy, I do not care how much calcium was in my body, I’m now taking calcium supplements, and even now, I’m sure I’ll take the iron tablet on time and every day.”(p.4).*

Another aspect of lifestyle modification was the effort to improve health behaviors extracted from the subcategories of attention to individual factors and environmental hazards. Increasing the accuracy of the model of behavior and increasing the observance of health issues was the focus of most participants. For example, two of the participants said:

*“In order not to allow microbe to enter my body, I wash my hands after doing all the work. In the meantime, I might have changed my clothes for two days, but now I change my underwear every day.”(p.3).*

*“I’m trying to take my monthly care in a timely manner, I could not have been careful about controlling my health previously.”(p.4).*

Regarding environmental hazards, the participants referred to environmental health, the use of protective equipment and efforts to ensure environmental safety. In this regard, two of them said:

*“I arrange the furniture and tools of the house from now in a way that we will be healthy, and also the child who will be born does not encounter them, so that he/she will not hurt.”(p.5).*

*“Now that the air is polluted, it’s worrying, so I’ll use mask from the beginning of pregnancy.”(p.6).*

Apart from the changes mentioned in the daily life, participants pointed to the change in their sleep and rest pattern extracted from the subcategories of attention to sleep hygiene and the need for further rest. In this regard, the participants stated:

*“I prefer to sleep on my left side, if it is not possible on the right side and not usually on my back” AND “Now (in pregnancy), I’m careful about the time of my sleeping, my sleep and my rest are regular now, according to the plan”(p.7).*

*“Everything I do, I’m resting. Pregnancy is not like normal situations. The energy is finished too early. I try to rest”. (p.6).*

### Adopting new behaviors

Adopting new behaviors was a main category extracted from sub-category of conducting behaviors adapted to new needs and preventive behaviors. Pregnant mothers adopt new behaviors to adapt to physical and emotional changes in pregnancy. According to pregnant women, pay more attention to their behaviors to prevent disease during pregnancy increases and most participants try to use supplements and medications, to avoid harmful effects during pregnancy, and to take measures to control weight gain in order to improve and promote their health. For example, some of them mentioned:

*“Now I’m not alone. If I was just myself, if I have a headache I’ll take sedative, but I cannot do it now. We must be careful not to get sick because the medicine cannot be consumed.”(p.6).*

*“I studied the Internet and books, and I made a diet for myself so that my weight will not be increased.”(p.2).*

*“Since my weight was already high, I’m now trying not to use high-fat foods; instead, I use vegetables and fruits.”(p.15).*

Avoiding harmful acts on health is another effort by pregnant women to promote their health; and a kind of physical self-care in pregnancy that was distance from the Internet and cell phones because of their radio waves as well as the lack of using cosmetics because of harmful substances were mentioned. For example, two subjects said that:

*“I eliminate WI-FI in pregnancy because of its damages.” (p.7).*

*“I do not use cosmetics and health products at all, because they can be absorbed to the body and may be harmful to the baby.”(p.8).*

Among other behavioral changes, attention was drawn to sexual behaviors that most pregnant women referred to decreased sexual activity and unwillingness to have sex and changing their sexual activity. For example, one of the participants said that:

*“Sexual intercourse has become lower, limited and more cautious, although stress is becoming more prominent in the sexual intercourse (laughs).” (p.9).*

### Mental self-care

This theme was classified into three main categories, including Stress management strategies, tendency to social interactions, and seeking support, which consists of six categories and fifteen subcategories.

### Stress management strategies

One of the measures stated by pregnant mothers was to drive disturbing thoughts and efforts to reduce stress, distracting, and trying to forget about problems was to fill leisure times and entertain. For example, two of the participants said that:

*“When some people bother me, especially my husband’s family (laughs), I do not pay attention to their words that hurt me, I turn around as if I did not hear.” (p.10).*

*“I like to go outside and go to the market with my sister, talk or walk. It is very influential in my spirit.”(p.3).*

Another aspect of stress coping strategies was the use of problem solving behaviors. Most participants talked about stress control factors and coping strategies, focusing on solving the problem, need for psychological counseling, avoiding confronting stressful people and environments, tolerating and confronting problems, and trying to control anger. For example, a pregnant woman said that:

*“I try to cut off the relationship with people who have a lot of anxiety and limit my relationship with such people.”(p.9).*

Other behaviors of women related to improving mental health included having positive thoughts, going towards spirituality and feeling of evolutionism. In this regard, the participants pointed out:

*“When I talk to my husband about our child, our hopes and motives for the future increase. I also believe in normal delivery. I’m ready to tolerate any pain to see my child.”(p.2).*

*“In the first pregnancy, I did not understand anything, but later I felt that we would become a big family, and felt maternal feeling.”(p.11).*

In terms of spirituality, most pregnant women tried to achieve mental relaxation and proximity to God through religious practices, enduring hardship, and correcting past moral defects; they also talked about having different feelings, such as being steady in life and subjecting God’s particular attention which suggests a sense of evolution. For example, two people said:

*“It is good to have communication with God during pregnancy and it can make God love you somehow. Very important, for example, the Qur’an will make you feel such a calmness as you no longer think about your matters.”(p.5).*

*“I try to participate in many religious ceremonies during my pregnancy.”(p.12).*

### Tendency to social interactions

The tendency towards social interactions is a main category including the subcategory of the tendency toward group membership. What mothers emphasized and loved was being alongside pregnant women and interacting with each other. Two of the participants said:

*“Participating in pregnancy classes is very good because they all are like you. I like that all are pregnant. I feel happy and convenient that everything can be said, we learn things from each other and it is good for our spirit.”(p.8).*

*“One should try to interact with colleagues and friends. Let’s go together to talk about a place. It has a lot of psychological effects. For example, let’s go to walking collectively. All these things have health effects.” (p13).*

### Seeking for support

Seeking for support is a main category, extracted from subcategories of psycho-emotional support, instrumental and information support. Pregnant mothers try to establish close relationships with family members and relatives, improve their relationship with their spouse and receive support for improving their health. It was also considered important to receive the financial and non-financial support for those around them. In this regard, the participants mentioned that:

*“Finally, I have another child. My husband can care him, so I can rest much. When I return from work, this is a kind of attention and help. I now love to be supported psychologically by hugging (laughing) with kissing.” “I’m trying to go to the house of my relatives. I visit them more and more. It’s good for the mood.”(p.14).*

On the other hand, gaining support from health care providers is important for pregnant mothers and they want to receive emotional support from care providers. For example, one of the mothers said:

*“I expect them to provide a good time to control my situation and explain me well.”(p.12).*

Regarding the instrumental support, one of pregnant women said that:

*“I would love that someone gives me present, for example, my sister, my friend, my husband, especially the books I need to read now.” (p.5).*

Another important aspect pregnant women seek to achieve in order to improve their health includes.

receiving information support from health care providers and supporting lawmakers /policymakers. For example, two subjects said that:

*“I ask my doctor step by step what I’m allowed to do this month, for example; traveling or exercise.”(p.10).*

*“Pregnant lady is not always pregnant. She is pregnant once for a certain time, it’s better to give pregnant women better facilities and the government helps her with costs.”(p.15).*

### Risk perception

This theme has two main categories of controlling the threat and perception the threat extracted from four subcategories and eight subcategories, respectively.

#### Perception the threat

this is a main category derived from the perception of pregnancy outcome and perception of the health sensitivity. What received from the interview was that pregnant mothers expressed their effort to improve their health due to their feeling of being at risk for the health of the fetus, as well as achieving the desired result of pregnancy and achieve more favorable and effective health strategy.

*“Well, if my weight is too much, my pressure will be too high, it’s not good for the baby, I’m worried about it; thus, I’ve completely eliminated salt.” (p.16).* In addition, a woman says, *“I always think that nothing happens for the baby; however, if it is the first baby, it is stressful.”(p.17).*

*“Anger is not good. It affects child’s ethics. Well, it affects baby’s health and also affects the ethics.”(p.16).*

#### Controlling the threat

Threat control is a major category extracted from the sub-category of trying to pass healthy pregnancy and reducing risk factors. Concerns about the future of the child and thinking that health-related behavior can lead to desirable outcomes make pregnant women try to change their behavior. Being serious and acting on health recommendations, as well as trying to control weight gain are actions effectively taken by pregnant mothers. For example, pregnant mothers stated that:

*“All of this is because to keep my child healthy and be efficient and independent in the society.”(p.5).*

*“Health improves the quality of life. A healthy person also has a healthy mind and a healthy life.” (p.5).*

*“It’s important for me to do things that make you healthy. To be healthy, be patient, and taking the work easy are the biggest motives.”(p.13).*

## Discussion

In this study, it was tried to examine the experiences of overweight pregnant women about healthy behaviors in Iran for the first time. The results of content analysis indicated that overweight pregnant women had a wide range of measures such as lifestyle modification, adopting new behaviors, Stress management strategies, tendency to social interactions and seeking social support in the form of physical and mental self-care in order to maintain and improve their health and achieve the desired outcome of pregnancy. The practice of these health-related behaviors is due to the perception of the threat and sense of threat control, which are related to their health status, and especially the health of their fetus. Some of these actions and behaviors are based on the background of life, in other words those can be influenced by habits, past learning and environmental culture [23, 31, 32]. As studies have reported that in different cultures, pregnant women act differently in health behaviors [23, 33], also in other study health literacy, education, and income levels have been reported to be effective in addressing health behaviors [5, 34]. But what is being addressed in this study is an overview of women’s efforts to make changes in various aspects of.

health behaviors.

In the study conducted by Baheiraei et al. in Iran, the experiences of health promotion behaviors in women of reproductive age have been studied. The main category extracted from the study include the proper food pattern, the creation of a proper rest and activity pattern, self-actualization, stress management, personal sensitivity and responsibility, the creation of a proper pattern of social interactions, healthy recreation exercises, and a sense of improving physical and functional health, as well as improvement of mental health which is consistent with the current study [19]. Although pregnant women have also taken new behaviors due to changes in pregnancy, they are also more sensitive to their own behaviors, who often discussed changing their behaviors compared to the pre-pregnancy time.

In the study of Higgens in the United States examining changes in health behaviors in pregnant women in the form of quantitative content analysis, pregnant women referred to changes in their diet, such as taking vitamins, frequent feeding with low amounts, increasing the consumption of vegetables and fruits. In addition, changes in activity patterns such as high walking and decreased heavy activity were mentioned. In this study, pregnant women reported changes in their habits of smoking, alcohol and caffeine use, regular check-ups, fluid intake, stress control efforts, hygiene observation, abdominal skin care and participation in pregnancy classes as the behavioral changes [35]. However most of the reported aspects of healthy behaviors are consistent with the present study, other aspects such as spirituality, social interactions, and healthy activities have not been mentioned for controlling stress.

Changes related to healthy behaviors in pregnant women over the age of 35 years in the study of Viau et al., including increased intake of fluids, reduced caffeine and alcohol use, and smoking, weight gain pattern control, folic acid consumption, avoidance of high-fat food intake, attention to nutrition and getting enough energy, changing daily activities, avoiding over-the-counter medications, and changing work and travel plans [36]. There are no other aspects of health promotion behaviors compared to the current study; while in our study, the aspects of self-care and efforts to achieve mental health were emphasized by pregnant women.

In this study, life-style modification was expressed in various aspects of lifestyle by pregnant women that, nutrition-related behaviors have been mentioned as one of the first changes in all interviews without exception. It is also mentioned in various studies [19, 23, 37].

Among the other behaviors that pregnant women referred to for maintaining and improving their health, there were modification of health behaviors and attention to personal hygiene and environmental risks; however, similar cases have been rarely mentioned in other studies, such as paying attention to frequent washing of hands, oral hygiene and sexual health. Efforts to avoid risk factors such as mobiles and radiation and cosmetics are among items that have not been addressed in other studies.

What is obvious from the evidence is that, change in sleep and rest patterns is of the usual changes although pregnant women try to do physical activity, such as walking; however, due to changes in pregnancy, the intensity and duration of physical activity is reduced compared to pre-pregnancy and they mentioned they need to rest in many cases. This indicates a not-so-good condition in terms of physical activity, which in other studies, an increase in resting time has been suggested by pregnant women [35, 37]. In some studies, according to women, diet has been more important than physical activity [4, 32]. However, in the present study, pregnant women referred to the details, how to change these behaviors, such as the creation of order, and changing the position of sleep.

In the present study, some changes in the behaviors created by women due to the changes and pregnancy needs have been expressed, such as the use of vitamins, the lack of arbitrary use of drugs, attempts to avoid becoming sick, attempts to control weight and not to carry out heavy activities that are consistent with other study. Nevertheless, compared to other studies, in the present study, pregnant women have also discussed the changes in sexual behavior during pregnancy.

Psychosocial self-care is a very important part of the present study; so that, an extensive range of measures was expressed to achieve mental relaxation and stress management by participants. These include Stress management strategies, social interactions and seeking social support. Although in quantitative studies, social support and stress have been associated with mental health status, such experiences have been addressed in less study. In the study of Baheiraei and et al., such measures have been mentioned from women of reproductive age, which can be due to cultural similarity [19].

In addition, as already mentioned, the change in the behavior adopted by pregnant women results from a perceived risk associated with the health of their fetus and themselves; and women are trying to achieve the best outcome of pregnancy. This finding can confirm the health belief model, based on which the individuals change their behaviors when they see their health at risk [38]. This finding is consistent with the results of other studies [23, 26, 36]. Only in the study of Sue et al., one of the participants has shown the tendency to have a proper appearance as a factor in the adoption of changes in nutrition and physical activity [26]. This study showed that pregnant mothers were eager to make positive changes in their everyday behaviors and they are looking for a safe way to ensure the child’s health; therefore, they are trying to use various sources to gain the necessary knowledge; and they want to benefit from the support of their health care providers.

The difference between our study and other studies is that we considered all aspects of health-related behaviors, while in other studies were addressed only a specific aspect of health promotion behaviors such as dietary patterns or type of physical activity. In this study, It is shown that pregnant women, in spite of their particular physical and mental status during pregnancy, are trying to change their behaviors in order to achieve the best outcomes, and therefore have different needs for other groups such as women of reproductive age, also, what should be considered is the individual and demographic differences in pregnant women such as the number of pregnancies, the level of literacy and employment status can affect how to adopt health behaviors, So these should be considered in planning of interventions.

### Strengths and limitations

This is the first study in Iran that examines the experiences of overweight pregnant women in relation to health promotion behaviors. However, like other qualitative studies, caution must be considered for generalizability; especially since most participants had high education level and were Primigravida.

## Conclusion

The findings of this study are in response to the question of what is the meaning of health promotion behaviors in pregnancy from the perspective of overweight pregnant women and what are its aspects, and create comprehensive view for researchers, give meaning to the concepts expressed as health promotion behaviors including a set of behavioral and cognitive actions resulting from motivation and risk perception in the overweight pregnant mother. It is in the form of self-care and strengthening the physical and mental health with the goal of having a healthy baby. Physical self-care activities are primarily associated with modifying the life-style during pregnancy and adopting new pregnancy-related behaviors and mental self-care behaviors in stress-coping strategies, social interactions and seeking social support. It can be said that pregnancy is an ideal opportunity for assessing behaviors and using the high motivation of women to guide them in adopting health behaviors and helping them follow health behaviors. Of course, for the proper interventions in this field, it is necessary to study the perception and view of the health care providers and the pregnant women’s relatives, especially their spouses about how mothers support the adoption of health behaviors.

## Abbreviations

BMI: Body Mass Index
